# Xanthine Derivative KMUP-1 Attenuates Experimental Periodontitis by Reducing Osteoclast Differentiation and Inflammation

**DOI:** 10.3389/fphar.2022.821492

**Published:** 2022-04-28

**Authors:** Cheng-Hsiang Kuo, Ban-Hua Zhang, Shang-En Huang, Jong-Hau Hsu, Yan-Hsiung Wang, Thi Tuyet Ngan Nguyen, Chao-Han Lai, Jwu-Lai Yeh

**Affiliations:** ^1^ International Center for Wound Repair and Regeneration, National Cheng Kung University, Tainan, Taiwan; ^2^ Graduate Institute of Medicine, College of Medicine, Kaohsiung Medical University, Kaohsiung, Taiwan; ^3^ Department of Pediatrics, Kaohsiung Medical University Hospital, Kaohsiung, Taiwan; ^4^ Department of Pediatrics, School of Medicine, Kaohsiung Medical University, Kaohsiung, Taiwan; ^5^ School of Dentistry, College of Dental Medicine, Kaohsiung Medical University, Kaohsiung, Taiwan; ^6^ Orthopaedic Research Center, College of Medicine, Kaohsiung Medical University, Kaohsiung, Taiwan; ^7^ Department of Medical Research, Kaohsiung Medical University Hospital, Kaohsiung, Taiwan; ^8^ Regenerative Medicine and Cell Therapy Research Center, Kaohsiung Medical University, Kaohsiung, Taiwan; ^9^ Cardiovascular Research Center, National Cheng Kung University, Tainan, Taiwan; ^10^ Department of Biochemistry and Molecular Biology, College of Medicine, National Cheng Kung University, Tainan, Taiwan; ^11^ Department of Surgery, National Cheng Kung University Hospital, College of Medicine, National Cheng Kung University, Tainan, Taiwan; ^12^ Department of Biostatistics, Vanderbilt University Medical Center, Nashville, TN, United States; ^13^ Department of Pharmacology, School of Medicine, Kaohsiung Medical University, Kaohsiung, Taiwan

**Keywords:** Porphyromonas gingivalis, lipopolysaccharide, inflammation, osteoclastogenesis, xanthine derivative, cGMP signalling

## Abstract

Periodontitis is an inflammatory disease of gum that may predispose to serious systemic complications such as diabetes and cardiovascular diseases. Activation of macrophages and osteoclasts around periodontal tissue can accelerate gum inflammation. In addition, alteration of cyclic nucleotide levels is associated with the severity of periodontitis. Our previous study has shown that KMUP-1, a xanthine derivative exhibiting phosphodiesterase inhibition and soluble guanylyl cyclase activation, can inhibit lipopolysaccharide (LPS)-induced inflammation and receptor activator of nuclear factor kappa-Β ligand (RANKL)-induced osteoclastogenesis. This study was aimed to investigate whether KMUP-1 could attenuate periodontitis both *in vitro* and *in vivo*. *In vitro*, the protective effect of KMUP-1 on inflammation and osteoclastogenesis was investigated in RANKL-primed RAW264.7 cells treated by *Porphyromonas gingivalis* LPS (*Pg*LPS). The results showed that KMUP-1 attenuated *Pg*LPS-induced osteoclast differentiation as demonstrated by decreased TRAP-positive multinuclear cells and TRAP activity. This reduction of osteoclast differentiation by KMUP-1 was reversed by KT5823, a protein kinase G inhibitor. Similarly, pro-inflammatory cytokine levels induced by *Pg*LPS were inhibited by KMUP-1 in a dose-dependent manner whereas reversed by KT5823. Mechanistically, suppression of MAPKs, PI3K/Akt, and NF-κB signaling pathways and decrease of c-Fos and NFATc1 expression in osteoclast precursors by KMUP-1 may mediate its protective effect. *In vivo*, two models of periodontitis in rats were induced by gingival injections of *Pg*LPS and ligature placement around molar teeth, respectively. Our results showed that KMUP-1 inhibited alveolar bone loss in both rat models, and this effect mediated at least partly by reduced osteoclastogenesis. In conclusion, our study demonstrated the therapeutic potential of KMUP-1 on periodontitis through suppression of inflammation and osteoclast differentiation.

## Introduction

Periodontitis is a common chronic disease characterized by inflammation of the gingiva and the surrounding structures of the periodontium (Terrizzi et al., 2013; How et al., 2016). Clinically, periodontitis has been shown to play a role in the pathogenesis of systemic disorders, including cardiovascular diseases, diabetes, and Alzheimer’s disease (How et al., 2016; Elwishahy et al., 2021; Pirih et al., 2021). Alveolar bone loss is one of the major hallmarks for its progression, and therefore preventing bone loss has been considered the key to periodontitis treatment (Hienz et al., 2015). Bone remodeling is a dynamic process maintained by a balance between bone formation and resorption (Suda et al., 1992; Miyazaki et al., 2011). In periodontitis, chronic inflammation disrupts the homeostatic bone metabolism in favor of bone loss (Sokos et al., 2015). In line with this, over-activation of osteoclast formation has been associated with periodontitis progression; therefore, interfering with inflammation and osteoclast differentiation has the potential to attenuate periodontitis.

Lipopolysaccharide (LPS) derived from *Porphyromonas gingivalis* (*Pg*LPS), can trigger gum inflammation which amplifies osteoclast differentiation and consequent alveolar bone absorption, thus plays an essential role in the pathogenesis of periodontitis (How et al., 2016; Sakanaka et al., 2016; Liljestrand et al., 2017; Zhang et al., 2020). Imbalanced bone remodeling in periodontitis elicited by *Pg*LPS has been attributed to increased osteoclast differentiation and decreased osteoblast generation (Pokhrel et al., 2017). Our previous study has shown that *Pg*LPS promotes pro-inflammatory cytokine expression, which in turn activates ERK and p38 pathways, thereby promoting macrophage polarization and osteoclast differentiation (Chang et al., 2021). In line with this, pro-inflammatory cytokines such as TNF-α and IL-6 can further accelerate osteoclast maturation (O'Brien et al., 2016; Chang et al., 2021; Sun et al., 2021).

Receptor activator of NF-κB (RANK) ligand (RANKL) is essential for osteoclast differentiation and recruitment (Suda et al., 1999). Activation of the NF-κB, MAPKs and PI3K/Akt pathways by RANKL is crucial for osteoclastogenesis (Leibbrandt and Penninger, 2008; Chen et al., 2018a). The RANKL/RANK pathway triggers interactions between TRAF6 and RANK (Alippe et al., 2017; Wu et al., 2017), activating the NF-κB and MAPKs pathway. Interactions between RANK and c-Src result in Akt phosphorylation and activation of the Akt pathway, triggering the release of intracellular Ca^2+^ and increase of nuclear NFATc1, a key transcription factor for osteoclast differentiation (Chen et al., 2018b). In addition to NFATc1, c-Fos downstream of JNK is important for osteoclast-macrophage lineage determination, sufficient osteoclast differentiation, and bone remodeling. In summary, inflammatory stimulation such as LPS could amplify osteoclastogenic signaling in part through enhancing RANK-mediated signaling and thus osteoclastogenesis progression.

KMUP-1, a unique xanthine and piperazine derivative developed by our group, 7-{2-[4-(2-chlorophenyl) piperazinyl] ethyl}-1,3-dimethylxanthine, exhibits profound inhibition on phosphodiesterases (PDE 3, 4, and 5), therefore increasing cellular level of cAMP and cGMP. Administration of KMUP-1 results in relaxation of smooth muscle in the aorta (Wu et al., 2001), corpus cavernosum (Lin et al., 2002) and trachea (Wu et al., 2004). In addition, KMUP-1 possesses pleiotropic properties, including anti-inflammation (Wu et al., 2006; Huang et al., 2021), anti-proliferation (Liu et al., 2009), cardio-protection (Yeh et al., 2010), neuro-protection (Hsu et al., 2010), anti-osteoporosis (Liou et al., 2013; Liou et al., 2015), anti-apoptosis (Wu et al., 2016), and reversion of Kv channel over-activation in pancreatic cells (Lee et al., 2018). However, the effects of KMUP-1 on periodontal disease remain unclear. Of note, alteration of cAMP and cGMP levels in human saliva has been inversely associated with the severity of periodontitis (Mashayekhi et al., 2005). In this study, we demonstrated that *in vitro,* KMUP-1 protected against *Pg*LPS-induced inflammation and osteoclast maturation through anti-osteoclastogenic and anti-inflammatory activities, and *in vivo*, it attenuated periodontitis in two rat models.

## Materials and Methods

### Materials and Reagents

Unless indicated, all chemicals were from Sigma-Aldrich (Saint Louis, MO, United States). *Pg*LPS was purchased from InvivoGen (San Diego, CA, United States). RANKL was purchased from R&D Systems. KMUP-1 was synthesized in our laboratory. ELISA kits for MCP-1 and IL-6 were purchased from R&D Systems. Nuclear and cytoplasmic fractionation was performed using NE-PER Nuclear and Cytoplasmic Extraction Kit (Pierce Biotechnology, Rockford, IL) according to the manufacturer’s instruction.

### Cell Culture

Murine macrophage cell line RAW264.7 (BCRC6001) was cultured in Dulbecco’s Modified Eagle Medium with 10% fetal bovine serum.

### Western Blot Analysis

For western blot analysis, cell lysates were collected using lysis buffer (0.1% SDS, 0.5% sodium deoxycholate, 1% NP-40 and protease inhibitor mixture; PMSF, aprotinin and sodium orthovanadate). Equal amounts of cell lysates were separated by SDS-polyacrylamide gel electrophoresis and transferred onto a polyvinylidene difluoride membrane. After blocking with 5% skim milk in PBS containing tween 20 (0.05%), the membrane was probed with a primary antibody overnight at 4°C followed by incubation with an HRP-conjugated secondary antibody (GeneTex, United States) for 1 h at room temperature. The signal was detected using Western ECL Substrate (Millipore, United States) with the charged-couple device (CCD) camera (MiniChemi-Chemiluminescence, Sage Creation Sciences, BioRiver, Co., Ltd., China). The immunoreactive bands were quantitatively determined using ImageJ software. Equal protein loading was confirmed by β-actin (GeneTex, United States) or Lamin B2 (Sigma, United States). Antibodies against p-ERK1/2, JNK, Akt, p-Akt, p-IκBα, p-p65, c-Fos and p-PI3K were purchased from Cell Signaling, United States. Antibodies against ERK1/2 and p-JNK were purchased from Upstate Biotechnology Inc., United States. Antibodies against p38 were purchased from Santa Cruz Biotechnology Inc., United States. Antibodies against p-p38 were purchased from Abcam, United Kingdom. Antibodies against PI3K and p65 were purchased from Millipore, United States. Antibody against NFATc1 was purchased from Taiclone, Taiwan.

### Experimental Protocol for Evaluation of KMUP-1 on *Porphyromonas gingivalis* Lipopolysaccharide-Induced Pre-osteoclast Inflammation and Differentiation

**FIGURE 1:**
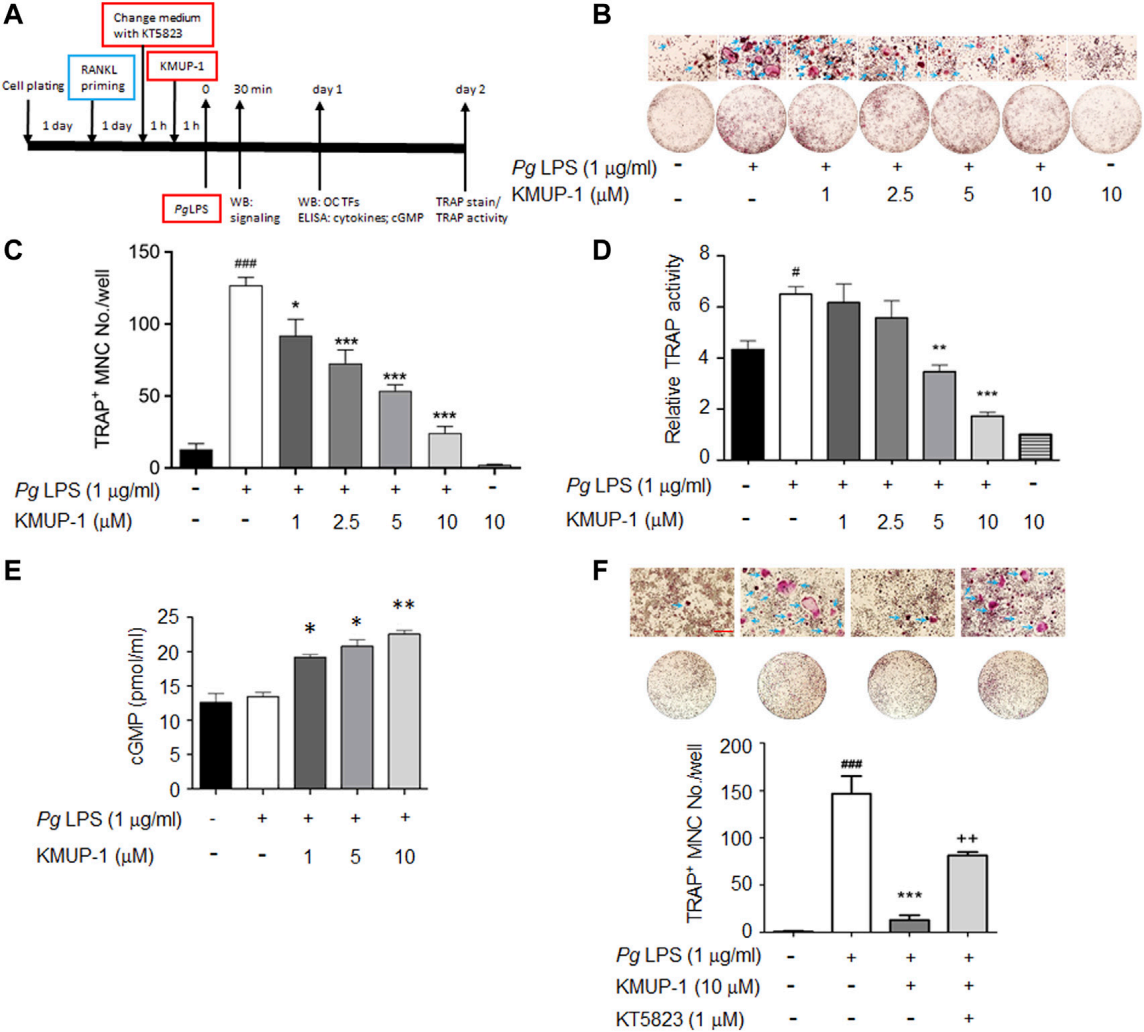
KMUP-1 attenuates osteoclast differentiation induced by *Pg*LPS in RANKL-primed RAW264.7 cells through PKG pathway. **(A)** Scheme of experimental protocol for evaluation of KMUP-1 on *Pg*LPS-induced osteoclast differentiation. RAW264.7 cells were pre-incubated with RANKL 1 day before *Pg*LPS stimulation in the presence of various concentrations of KMUP-1 which was applied 1 h before *Pg*LPS. KT5823 was applied 1 h before KMUP-1 when studying the effect of the cGMP-PKG pathway, otherwise RANKL-containing medium was replaced by KMUP-1-containing medium. **(B)** The representative images of TRAP stain with high power views on top row. Arrows indicate TRAP positive multinucleated cells (TRAP^+^ MNCs). **(C)** Statistical analysis of TRAP^+^ MNC number from **(B)**. **(D)** TRAP activity assay. **(E)** Elevation of cGMP level by KMUP-1. **(F)** KT5823 reverses KMUP-1’s effect on suppression of osteoclast differentiation induced by *Pg*LPS as demonstrated by TRAP stain. Scale bars: 100 μm. Data are the mean ± SD of three-independent experiments, with more than duplicate were performed in each experiment. #*p* < 0.05, ###*p* < 0.001 compared with control; *p < 0.05, **p < 0.01, ***p < 0.001 compared with *Pg*LPS; ++*p* < 0.01 compared with *Pg*LPS + KMUP-1.

The experimental protocol is summarized in Figure 1A. Briefly, 1 day after cell plating RAW264.7 cells were pre-incubated with RANKL (10 ng/ml) for 1 day before *Pg*LPS stimulation in the presence of various concentrations of KMUP-1, which was applied 1 h before *Pg*LPS (1 μg/ml). KT5823 was applied 1 h before KMUP-1 when studying the effect of the cGMP-PKG pathway; otherwise, RANKL-containing medium was replaced by KMUP-1-containing medium. To measure cGMP level 1 day after *Pg*LPS stimulation, a cGMP ELISA kit based on the competitive-ELISA principle was used following the manufacturer’s instructions (#E4717-100, BioVision, United States). Pro-inflammatory signaling and expression of osteoclast master transcriptional factors were assessed 30 min and 1 day after *Pg*LPS stimulation using western blot assay, respectively. The levels of MCP-1 and IL-6 in the supernatant were measured 1 day after *Pg*LPS stimulation with ELISA kits (R&D Systems, United States) according to the manufacturer’s instructions.

### Osteoclast Formation and Evaluation—Tartrate-Resistant Acid Phosphatase Activity Assay and Tartrate-Resistant Acid Phosphatase Stain

Two days after *Pg*LPS stimulation, the cells were washed with PBS and fixed with 10% paraformaldehyde solution for 10 min, followed by replacement with 95% alcohol. TRAP activity was measured by spectrophotometry using a 50 mM citrate buffer containing 10 mM sodium tartrate and 3.7 mM 4-nitrophenyl phosphate in a 37°C incubator for 30 min, and finally with 0.1 M NaOH to stop the reaction. The light absorbance at 405 nm was measured using a spectrophotometer. A TRAP staining kit (Sigma, United States) was used to analyze TRAP expression. The TRAP-positive multinucleated cells (TRAP^+^ MNCs) that contained three or more nuclei were counted as osteoclasts using optical microscopy.

### Experimental Periodontitis

Male Wistar rats (150–200 g) purchased from the Bio LASCO Taiwan Co., Lt., were used in this study. The experiments were conducted following the ARRIVE (Animal Research: Reporting of *in vivo* Experiments) guidelines and approved by the Kaohsiung Medical University animal center (Kaohsiung, Taiwan, IACUC Approval No. 108118). Rats were allowed free access to food and water ad libitum during the acclimatization and experimental period. The rats were kept in a good ventilated room at the standard experimental condition with room temperature (22 ± 2°C) and relative humidity (60 ± 10%) with a 12 hour-light/dark cycle. The experiment started after 1 week of adaptive feeding. For gingival injections of *Pg*LPS, the experiment was conducted as the previous report with some substantial modification (Elburki et al., 2014). Experimental animals were randomly divided into four groups: 1) Sham group received PBS, 2) *Pg*LPS group received 10 mg/ml *Pg*LPS, 3) *Pg*LPS + KMUP-1 group received 10 mg/ml *Pg*LPS + 10 mg/kg KMUP-1 and 4) KMUP-1 group received 10 mg/kg KMUP-1. Gum inflammation was induced by injection of *Pg*LPS into the palatal gingiva between the first and second maxillary molars on both sides. The *Pg*LPS was administrated once every 2 days, and KMUP-1 was given daily through oral administration for 3 weeks. All experimental animals were weighed every other day, and the dosage was adjusted according to their body weight. The ligature model was conducted as previously described with some modifications (de Molon et al., 2018). Briefly, the experimental rats were randomly divided into four groups: 1) Sham group received PBS, 2) ligature group received PBS, 3) ligature + KMUP-1 group received 1 mg/kg KMUP-1 and 4) KMUP-1 group received 1 mg/kg KMUP-1. In this model, ligatures were placed around second molar teeth, and KMUP-1 was applied through intraperitoneal injection daily for 30 days.

### Micro-Computed Tomography

A high-resolution micro-CT system (Skyscan 1,076) was used to scan the fixed maxilla samples. The voxel size was 1.0 mm, and the X-ray energy was set at 75 kV and 134 μA. NRecon Reconstruction Software, 3D Visualization Software, and CT Analysis Software were used to analyze three-dimensional structure and bone parameters.

### Histology and Tartrate-Resistant Acid Phosphatase Histochemical Staining

After experimental periodontitis assay, the experimental rats were sacrificed by Zoletil overdose for tooth harvest at indicated time points. Teeth and maxillae of rats were fixed in 10% paraformaldehyde solution with 0.5 M EDTA. The paraffin-embedded tissue section with 5 μm thickness was used for histology. A TRAP staining kit (Sigma, United States) was used to analyze TRAP expression according to the manufacturer’s instructions. The number of osteoclasts in the tissue section was evaluated by counting the TRAP^+^ cell numbers and normalized to the bone area.

### Statistical Analysis

Data are presented as mean ± standard deviation (mean ± SD). For comparisons between 2 groups, Student’s *t*-test was used. For comparisons between multiple groups, one way-ANOVA followed by Tukey post hoc test was used. A value of *p* < 0.05 is considered statistically significant.

## Results

### KMUP-1 Attenuates Osteoclast Differentiation Induced by *Porphyromonas gingivalis* Lipopolysaccharide in Receptor Activator of NF-κB Ligand-Primed RAW264.7 Cells Through PKG Pathway

Inflammation exacerbates periodontitis progression via accelerating osteoclast precursor differentiation which may be attenuated by anti-inflammatory KMUP-1. Since RANKL stimulation has been shown to induce macrophage polarization and thus exhibits a tendency to differentiate into osteoclasts (Huang et al., 2017; Chang et al., 2021), we used RANKL-primed RAW264.7 cells to assess the hypothesis above (Figure 1A). Indeed, *Pg*LPS significantly increased the formation of TRAP^+^ MNCs in RANKL-primed RAW264.7 cells while reduced in the presence of KMUP-1 (Figures 1B,C). Consistently, TRAP activity in *Pg*LPS-stimulated osteoclasts was reduced by KMUP-1 in a dose-dependent manner (Figure 1D). Since the elevation of cGMP level by KMUP-1 contributes to its cellular functions (Wu et al., 2001; Wu et al., 2006; Yeh et al., 2010), we analyzed if cGMP is involved in its protective effect on *Pg*LPS-induced osteoclast differentiation (Figure 1A). The cGMP level was not changed by *Pg*LPS but increased by KMUP-1 in a dose-dependent manner (Figure 1E). Using a PKG inhibitor, KT5823, we demonstrated that inhibition of *Pg*LPS-induced osteoclast differentiation by KMUP-1 was revered (Figure 1F). These results indicate that KMUP-1 could suppress *Pg*LPS-induced osteoclast differentiation partly through a cGMP-PKG-dependent pathway.

### KMUP-1 Attenuates Expression of *Porphyromonas gingivalis* Lipopolysaccharide-Induced Inflammatory Cytokines in Receptor Activator of NF-κB Ligand-Primed RAW264.7 Cells Through PKG

**FIGURE 2:**
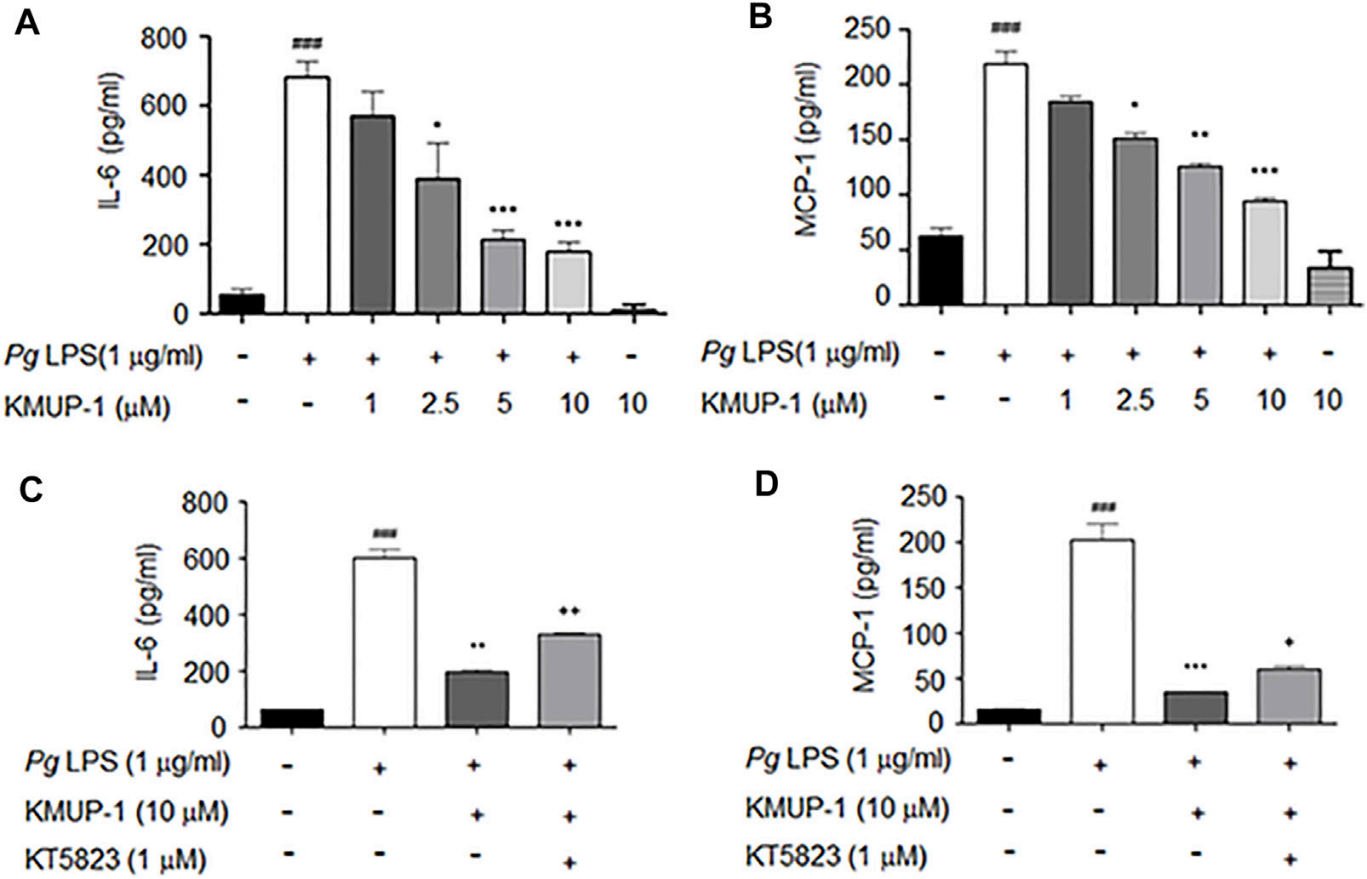
KMUP-1 attenuates *Pg*LPS-induced inflammatory cytokines in pre-osteoclasts through PKG. The expression of inflammatory mediators in culture supernatants were measured 1 day after *Pg*LPS stimulation in RANKL-primed RAW264.7 cells. **(A to D)** ELISA assay of proinflammatory cytokine expression. KMUP-1 dose-dependently attenuates **(A)** IL-6 and **(B)** MCP-1 expression. Blocking PKG using KT5823 reverses KMUP-1 on suppressing **(C)** IL-6 and **(D)** MCP-1 expression. Data are the mean ± SD of three-independent experiments, with more than duplicate were performed in each experiment. ###*p* < 0.001 compared with control; *p < 0.05, **p < 0.01, ***p < 0.001 compared with *Pg*LPS; +*p* < 0.05, ++*p* < 0.01 compared with *Pg*LPS + KMUP-1.

Since PKG and KMUP-1 all exhibit anti-inflammatory activity in macrophages (Mezzasoma et al., 2020; Huang et al., 2021), we investigated if KMUP-1 could modulate pro-inflammatory cytokine expression in pre-osteoclasts with *Pg*LPS stimulation. The expression of IL-6 (Figure 2A) and MCP-1 (Figure 2B) were drastically increased by *Pg*LPS while significantly reduced by KMUP-1 in a dose-dependent manner. Further, we investigated if suppression of inflamed osteoclast phenotypes by KMUP-1 is mediated with the PKG pathway using KT5823. Consistent with results demonstrated in osteoclast formation and differentiation (Figure 1), KT5823 significantly reversed the reduction of IL-6 (Figure 2C) and MCP-1 (Figure 2D) by KMUP-1 treatment. Taken together, these results indicate that inflammation in pre-osteoclasts was increased by *Pg*LPS, which can be attenuated by KMUP-1 in part through the PKG-dependent pathway.

### KMUP-1 Attenuates Pro-inflammatory Signaling Induced by *Porphyromonas gingivalis* Lipopolysaccharide

**FIGURE 3:**
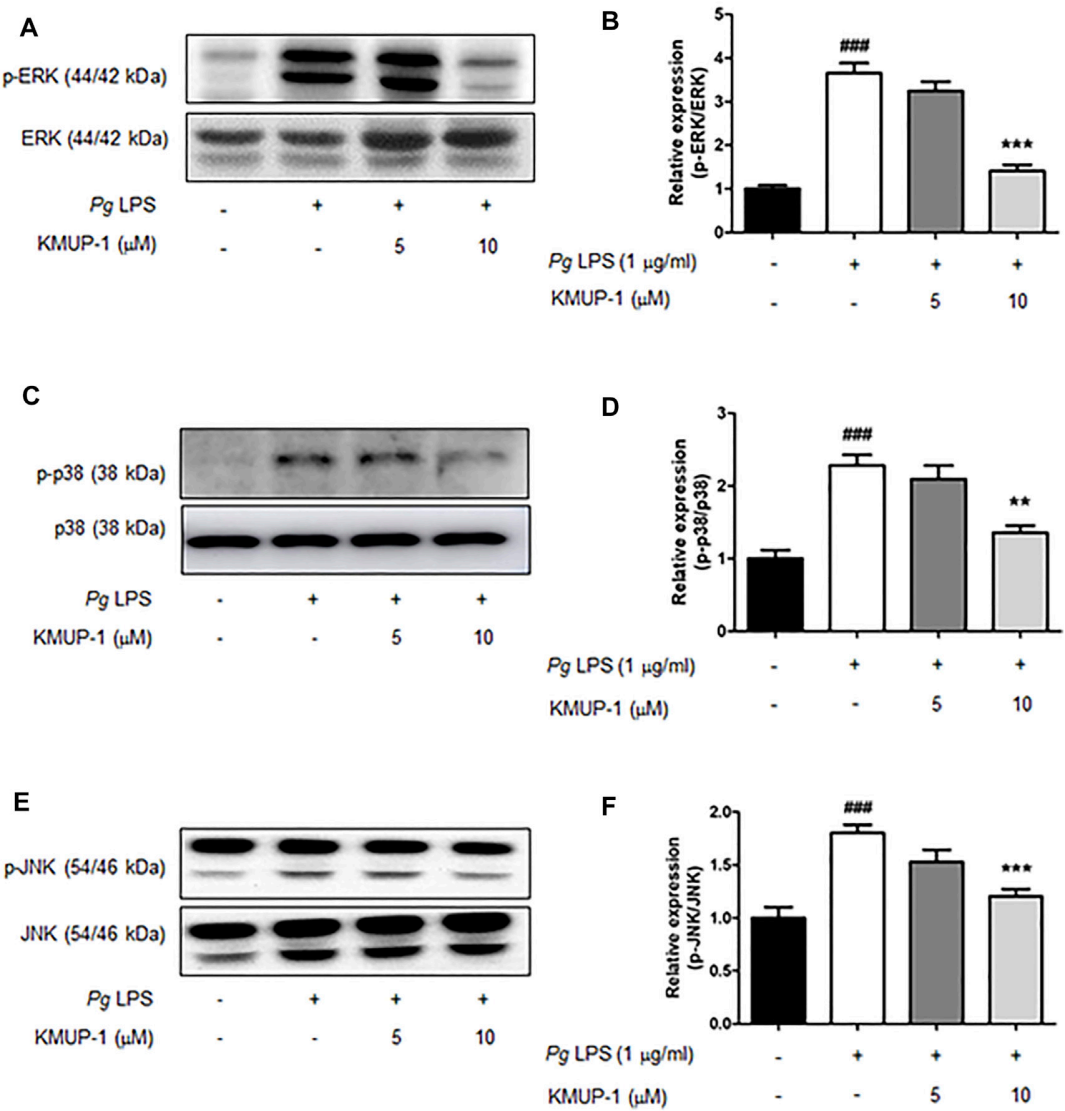
KMUP-1 attenuates pro-inflammatory signaling (MAPKs signaling) induced by *Pg*LPS in RANKL-primed pre-osteoclasts. The expression of pro-inflammatory mediators including **(A,B)** p-ERK/ERK, **(C,D)** p-p38/p38, and **(E,F)** p-JNK/JNK was analyzed in RANKL-primed pre-osteoclasts 30 min after *Pg*LPS stimulation using western blot assay. n = 5. ###*p* < 0.001 compared with control; **p < 0.01, ***p < 0.001 compared with *Pg*LPS.

**FIGURE 4:**
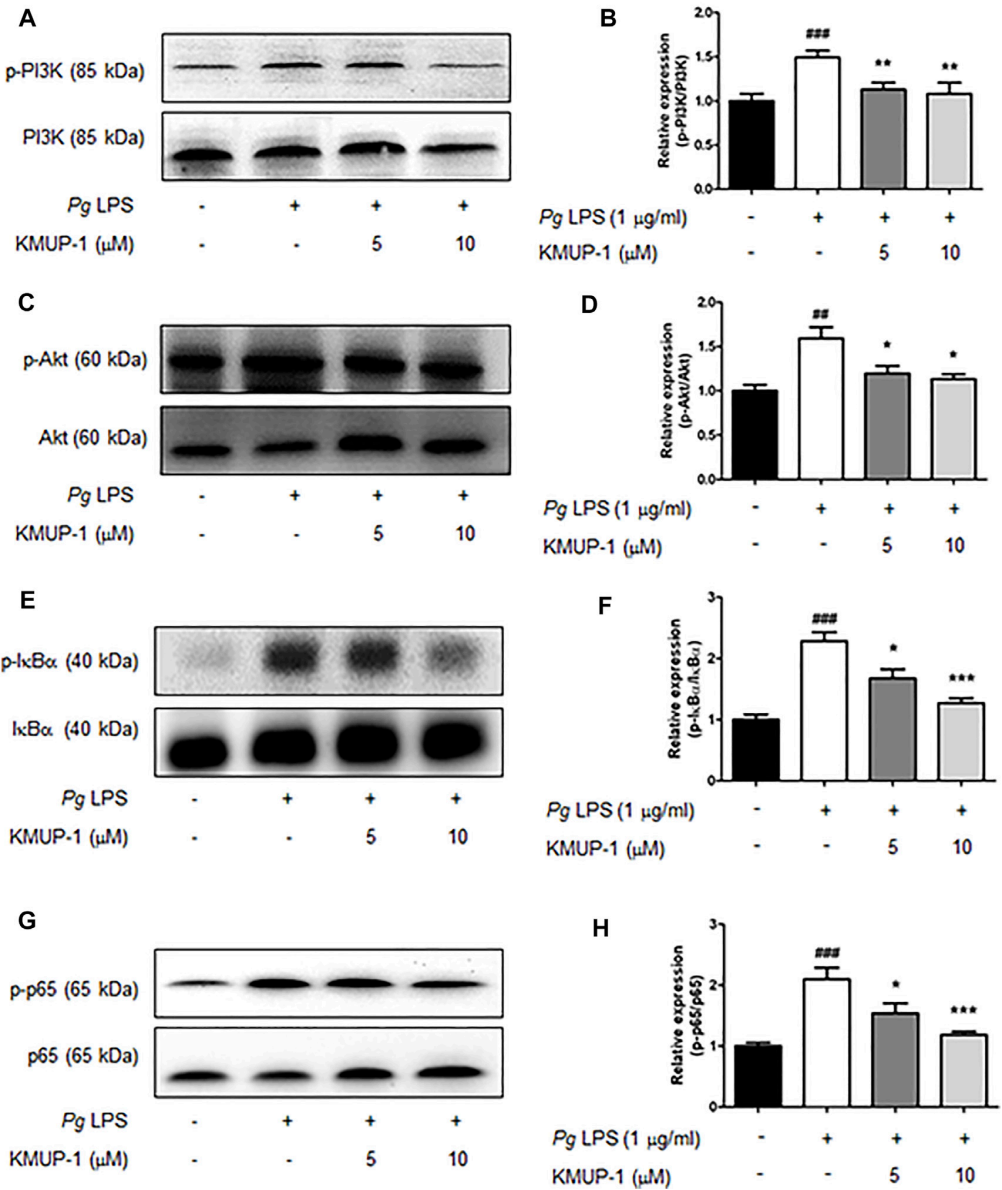
KMUP-1 attenuates pro-inflammatory signaling (Akt and NFκB signaling) induced by PgLPS in RANKL-primed pre-osteoclasts. The expression of pro-inflammatory mediators including **(A,B)** p-PI3K/PI3K, **(C,D)** p-Akt/Akt, **(E,F)** p-IκBα/IκBα, and **(G,H)** p-p65/p65 was analyzed in RANKL-primed RAW 264.7 cells 30 min after *Pg*LPS stimulation using western blot assay. n = 5. ##*p* < 0.01, ###*p* < 0.001 compared with control; *p < 0.05, **p < 0.01, ***p < 0.001 compared with *Pg*LPS.

We further analyzed the molecular mechanism underlying the suppression of KMUP-1 on *Pg*LPS-induced inflammatory osteoclasts. Activation of MAPKs, including ERK, p38, JNK, PI3K-Akt and NF-κB signaling pathways, have been involved in inflammatory responses induced by LPS (Teramachi et al., 2017; AlQranei and Chellaiah, 2020; Huang et al., 2021). We investigated if KMUP-1 treatment can change those signaling pathways in RANKL-primed pre-osteoclasts with *Pg*LPS stimulation. Indeed, *Pg*LPS significantly induced activation of MAPKs, including ERK (Figures 3A,B), p38 (Figures 3C,D), and JNK (Figures 3E,F) as well as PI3K (Figures 4A,B)-Akt (Figures 4C,D) pathways. Also, NF-κB signaling was activated, as evidenced by increased phospho-IκB (Figures 4E,F) and phospho-p65 (Figures 4G,H). With KMUP-1 treatment, all these pathways elicited by *Pg*LPS were significantly suppressed. These results further emphasize that anti-inflammatory KMUP-1 may suppress inflammatory gum disease.

### KMUP-1 Attenuates Expression of Master Transcription Factors for Osteoclast Differentiation Induced by *Porphyromonas gingivalis* Lipopolysaccharide

**FIGURE 5:**
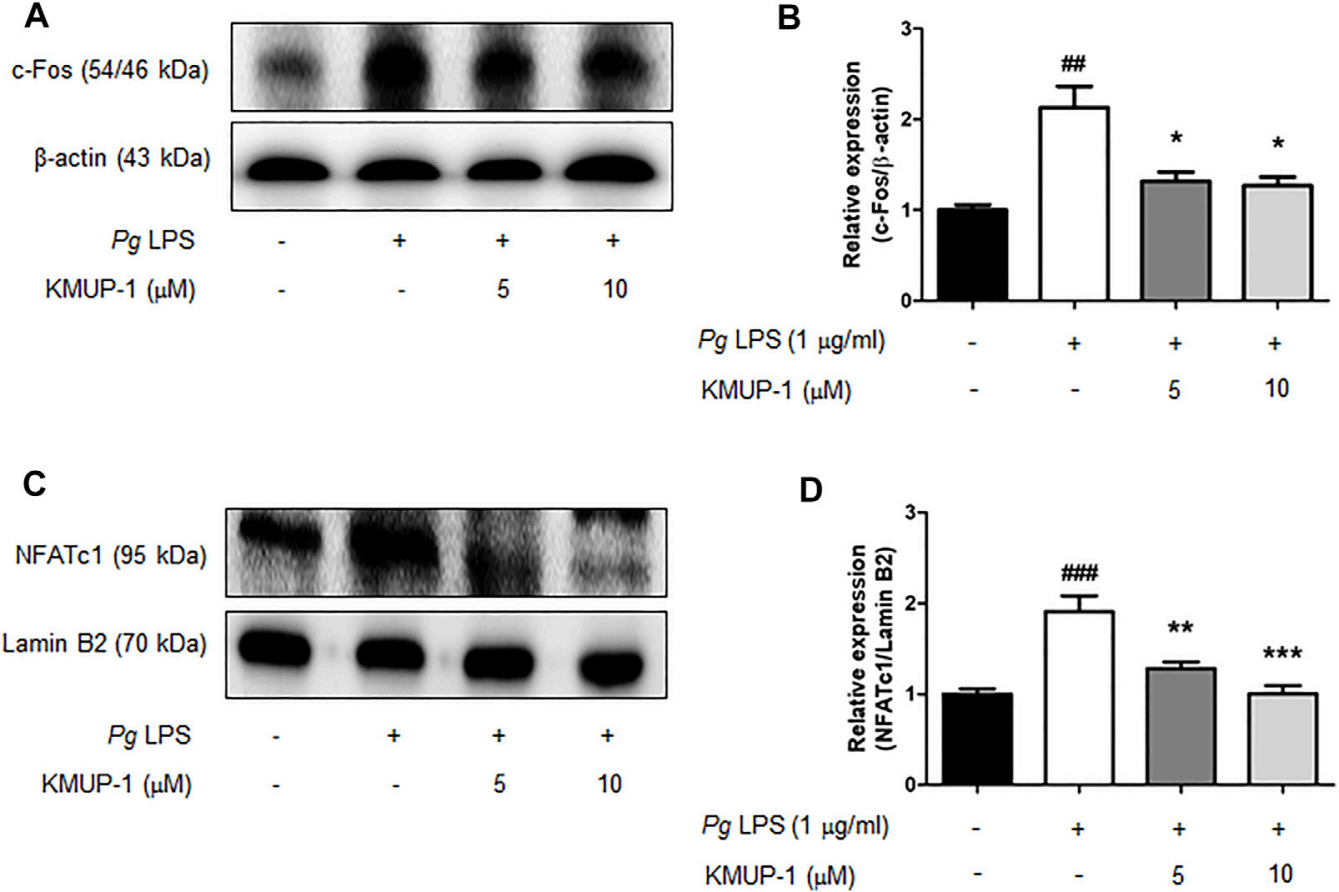
KMUP-1 attenuates c-Fos and nuclear NFATc1 expression induced by *Pg*LPS in RANKL-primed pre-osteoclasts. The expression of **(A,B)** c-Fos and **(C,D)** nuclear NFATc1was analyzed in RANKL-primed RAW 264.7 cells 24 h after *Pg*LPS stimulation using western blot assay. Lamin B2 or β-Actin was used as an internal control. n = 5. ##*p* < 0.01, ###*p* < 0.001 compared with control; *p < 0.05, **p < 0.01, ***p < 0.001 compared with *Pg*LPS.

Pro-inflammatory signaling pathways leading to osteogenic gene expression have been linked to osteoclastogenesis (Kodama and Kaito, 2020; Chang et al., 2021). Our results indicated that KMUP-1 can suppress *Pg*LPS-induced pro-inflammatory signaling pathways, cytokine expression, and osteoclast differentiation. Further, we investigate if KMUP-1 alters the expression of master transcription factors for osteoclastogenesis. Consistently, our results show that c-Fos (Figures 5A,B) and NFATc1 (Figures 5C,D) protein expression levels were significantly increased by *Pg*LPS while decreased by KMUP-1, suggesting that KMUP-1 may protect against periodontitis.

### KMUP-1 Protects Against Periodontitis Through Inhibition of Osteoclast Differentiation

**FIGURE 6:**
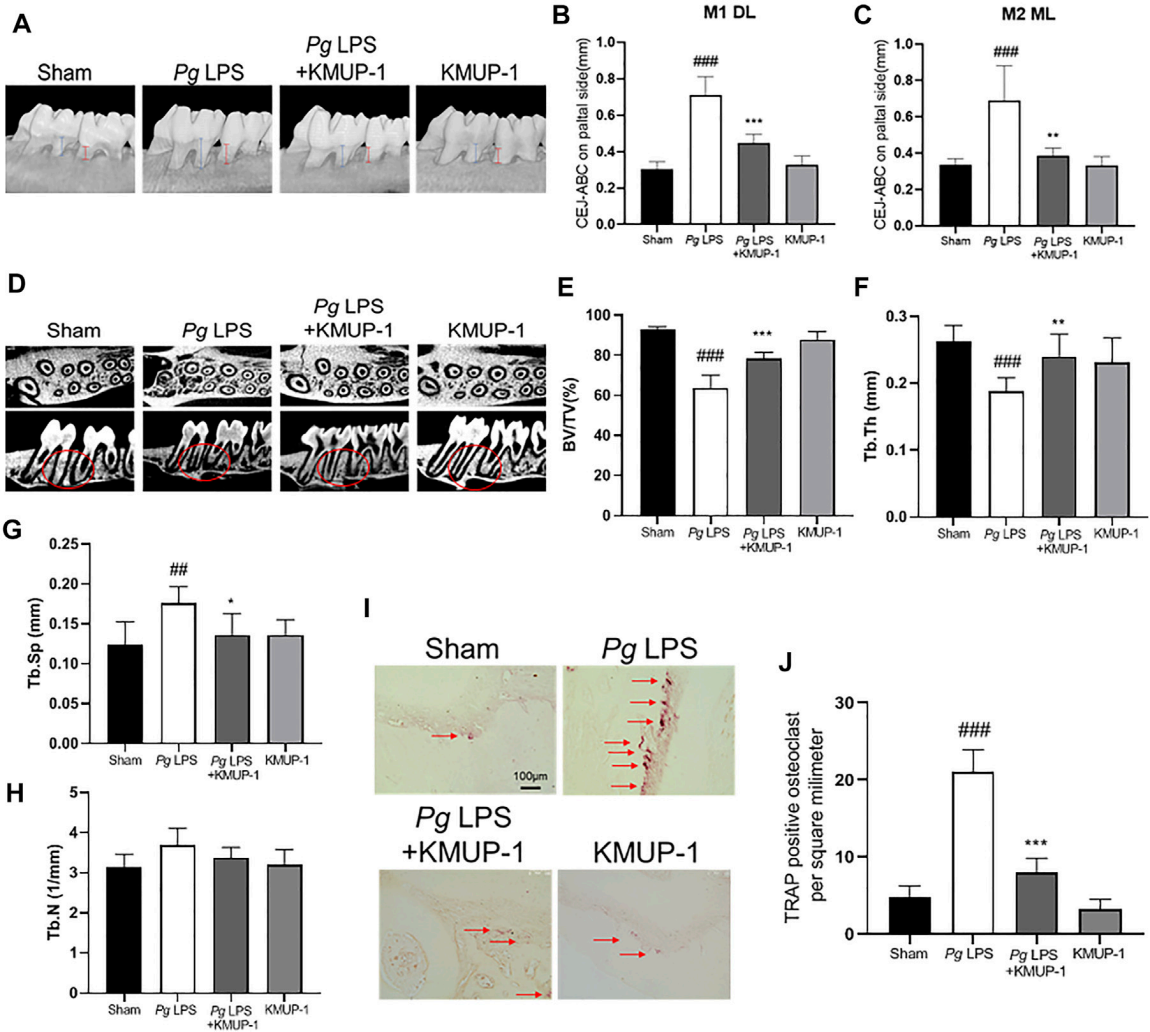
KMUP-1 protects against periodontal bone loss induced by *Pg*LPS. The periodontitis was induced by repeated injection of *Pg*LPS into gingiva. **(A)** Representative micro-CT images of palatal maxilla of experimental groups. The distance between cementoenamel junction (CEJ) to alveolar bone crest (ABC) at **(B)** first maxillary molar (M1, as denoted by blue lines) of distolingual (DL) and **(C)** second maxillary molar (M2, as denoted by red lines) of mesiolingual (ML) was measured 3 weeks after KMUP-1 treatment. **(D)** Representative longitudinal- and cross-sectional micro-CT images of the first and second molar in the maxilla. Circles indicate region of interest. The volumetric parameters, including **(E)** bone volume/tissue volume (BV/TV), **(F)** trabecular thickness (Tb.Th), **(G)** trabecular separation (Tb.Sp), and **(H)** trabecular number (Th.N) were measured. **(I)** Representative image of TRAP stain in maxillary alveolar bone sections. Arrows indicate TRAP^+^ cells. **(J)** Statistical analysis of osteoclast (TRAP^+^ cells) density in the maxillary alveolar bone. Scale bars: 100 μm n = 6. ##*p* < 0.01, ###*p* < 0.001 compared with sham. **p < 0.01, ***p < 0.001 compared with *Pg*LPS.

**FIGURE 7:**
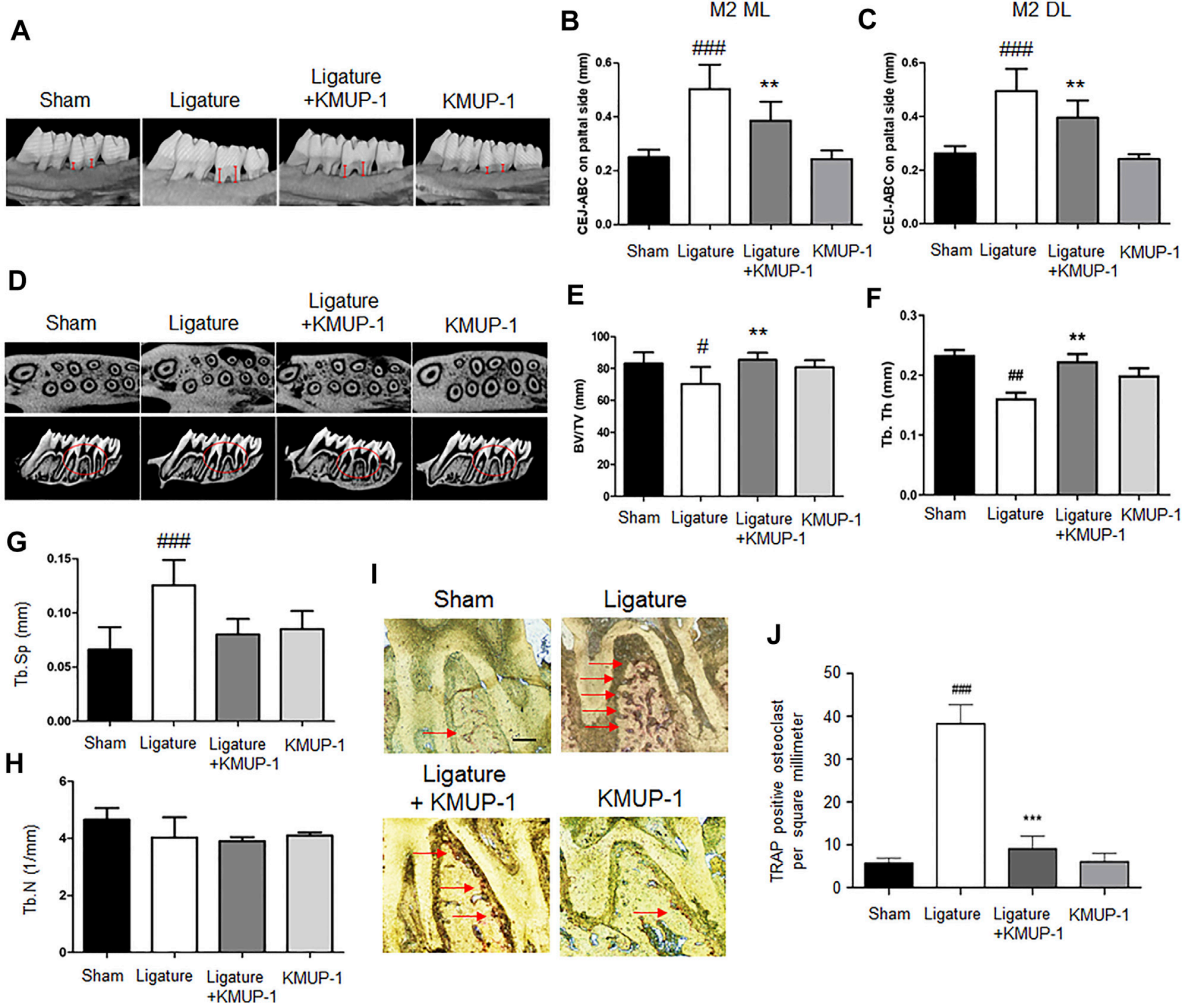
KMUP-1 protects against periodontal bone loss induced by ligature placement. The periodontitis was induced by placement of ligature around the cervical region of the maxilla molar. **(A)** Representative micro-CT images of palatal maxilla of experimental groups. The distance between cementoenamel junction (CEJ) to alveolar bone crest (ABC) at **(B)** second maxillary molar (M2) of distolingual (DL) and **(C)** second maxillary molar (M2) of mesiolingual (ML) was measured (as denoted by red lines) 1 month after KMUP-1 treatment. **(D)** Representative cross-sectional micro-CT images of the first and second molar in the maxilla. Circles indicate region of interest. The volumetric parameters, including **(E)** bone volume/tissue volume (BV/TV), **(F)** trabecular thickness (Tb.Th), **(G)** trabecular separation (Tb.Sp), and **(H)** trabecular number (Th.N) were measured. **(I)** Representative image of TRAP stain in the maxillary alveolar bone sections. Arrows indicate TRAP^+^ cells. **(J)** Statistical analysis of osteoclast (TRAP^+^ cells) density in the maxillary alveolar bone. Scale bars: 100 μm n = 6. #*p* < 0.05, ##*p* < 0.01, ###*p* < 0.001 compared with sham. **p < 0.01, ***p < 0.001 compared with ligature.

Since KMUP-1 exhibits superior effects on preventing inflammatory osteoclast formation, we evaluated its effect *in vivo* using rat models of periodontitis. In the *Pg*LPS injection model, the distance from the cementum-enamel junction to the alveolar bone crest (CEJ-ABC) of the maxillary first and second molars was significantly increased in the *Pg*LPS injection group, manifesting successful periodontitis induction (Figures 6A–C). Treatment with KMUP-1 effectively decreased *Pg*LPS-induced alveolar bone resorption (Figures 6A–C). The alteration of the bone structure by *Pg*LPS stimulation and KMUP-1 treatment was revealed by micro-CT examination (Figure 6D). Specifically, the decrease of the ratio of bone volume/tissue volume (Figure 6E) and trabecular thickness (Figure 6F) accompanied by an increase in trabecular separation (Figure 6G) upon *Pg*LPS injection was observed, whereas treatment of KMUP-1 significantly attenuated the abovementioned pathologic phenotypes of bone loss in *Pg*LPS-induced periodontitis model (Figures 6A–G). No significant changes in the trabecular number were observed among the groups (Figure 6H). Histologically, osteoclast number in the maxillary alveolar bone was significantly increased in the *Pg*LPS group while reduced by KMUP-1 treatment (Figures 6I,J). Moreover, ligature placement around molar teeth was conducted to further evaluate the effect of KMUP-1 on the suppression of periodontitis (Figure 7). The results show that periodontitis was induced by placement of ligature as demonstrated by the increase of CEJ-ABC distances (Figures 7A–C) and alteration in bone structures (Figures 7D–G). No significant change in trabecular number was observed (Figure 7H). The increase of osteoclast number in the maxillary alveolar bone by ligature placement was significantly reduced by KMUP-1 treatment (Figures 7I,J). In summary, our results demonstrated that KMUP-1 effectively suppressed experimental periodontitis in rat models, suggesting inhibition of osteoclast differentiation by KMUP-1 as observed *in vitro* may contribute to its protective function against periodontitis. These results indicated that KMUP-1 treatment protects against periodontitis bone loss and preserves trabecular structure.

## Discussion

Periodontitis is a chronic inflammatory disease resulting in the destruction of periodontal tissues (Dentino et al., 2013). Periodontal soft tissue inflammation and progressive loss of periodontal ligament and alveolar bone are major manifestations of periodontitis (Hajishengallis, 2015). Pathogenic bacteria accumulation, initially around the gingival margin and then into the periodontal pocket, has long been considered the cause of gingiva inflammation. *Pg*LPS has been considered one of the primary triggers for periodontitis (Kadono et al., 1999; McGraw et al., 1999; Kadowaki et al., 2000). KMUP-1, a xanthine derivative with phosphodiesterase inhibitor activity and anti-inflammation function, has been shown to modulate bone metabolism by enhancing osteoblastogenesis and inhibiting osteoclastogenesis in our previous studies (Liou et al., 2013; Liou et al., 2015). The current study demonstrates that KMUP-1 diminishes *Pg*LPS-mediated inflammatory osteoclast phenotypes and experimental periodontitis in rat models.

The balance between guanylate cyclase and phosphodiesterases controls cellular cGMP level (Pasmanter et al., 2021). Several lines of evidence implicate that cyclic nucleotides levels are associated with inflammation and bone homeostasis/metabolism (Holliday et al., 1997; Yamagami et al., 2003). In line with these observations, the anti-inflammatory function of cGMP/PKG has been proposed to interfere with inflammasome activation enhanced by LPS in macrophages (Mezzasoma et al., 2020). In addition, alteration of cAMP and cGMP levels in saliva is correlated with severe progression of periodontitis in humans (Mashayekhi et al., 2005). Of note, KMUP-1 modulates cyclic nucleotides level in several aspects of cellular functions (Lin et al., 2002; Wu et al., 2004; Wu et al., 2006; Yeh et al., 2010; Liou et al., 2013; Liou et al., 2015; Lee et al., 2018). For example, KMUP-1 reverses glucotoxicity-activated Kv channels through the cAMP/protein kinase A (PKA) signaling pathway in pancreatic beta cells (Lee et al., 2018). KMUP-1 inhibits tracheal smooth muscle cell inflammation through the cGMP/PKG but not cAMP/PKA signaling pathway (Wu et al., 2006). In addition, KMUP-1 suppresses RANKL-induced osteoclastogenesis and prevents ovariectomy-induced bone loss through both cAMP/PKA- and cGMP/PKG-dependent signaling pathways (Liou et al., 2013). Previously, we have demonstrated that RNAKL-induced osteoclastogenesis and the expression of osteoclastogenic genes in RAW264.7 cells are reduced by KMUP-1 while reversed in the presence of inhibitor for PKG (KT5823) or PKA (H89), indicating that the elevation of cAMP and cGMP by KMUP-1 is responsible for its protective effects. However, the effect of KMUP-1 on RANKL/*Pg*LPS-mediated inflammatory osteoclast phenotypes has not been investigated yet, especially in periodontitis. The current study demonstrated that *Pg*LPS did not alter the cGMP level in RANKL-primed pre-osteoclasts while KMUP-1 treatment significantly increased the cGMP level. Both the cAMP/PKA and cGMP/PKG signaling pathways may be involved in anti-inflammatory and anti-osteoclastogenic reactions elicited by KMUP-1 treatment in inflammatory gum diseases. In agreement with this, the reversion of KMUP-1-suppressed osteoclastogenesis by inhibition of PKG emphasizes that cGMP elevation is necessary for the protective function of KMUP-1.

RANKL/RANK pathway triggers osteoclast differentiation through various downstream signaling pathways, including TRAFs, MAPKs, PI3K-Akt, NF-κB, nitric oxide pathways, leading to activation of osteoclast precursors and osteoclastogenic gene expression (Galibert et al., 1998; Darnay et al., 1999; Inoue et al., 2000; Ross, 2006; Wada et al., 2006; Leibbrandt and Penninger, 2008). NF-κB and c-Fos regulate NFATc1 expression, thereby controlling the expression of osteoclast-specific genes, including TRAP, cathepsin K, and MMP-9 during osteoclast differentiation (Asagiri and Takayanagi, 2007; Nakashima and Takayanagi, 2011). In the case of LPS-induced osteoclastogenesis, TLR4/TARP6 pathway has been shown to elicit osteoclast formation and bone loss accompanied with activation of JNK, ERK, and p38 (He et al., 2016; Teramachi et al., 2017). KMUP-1 reduces inflammation and hyperalgesia in a bilateral chronic constriction injury model via suppressing MAPKs and NF-κB activation (Dai et al., 2014). In addition, KMUP-1 attenuates RANKL-induced macrophage transdifferentiation into osteoclasts and prevents ovariectomy-induced bone loss through inhibiting MAPKs, Akt, NF-κB and calcium/calcineurin/NFATc1 pathways (Liou et al., 2013). Recently, we also demonstrated that KMUP-1 reduces LPS-induced MAPKs and NF-κB activation in part through induction of SIRT1 in macrophage and protects against cartilage erosion in a rat model of osteoarthritis (Huang et al., 2021). In the current study, we demonstrated that *Pg*LPS activates MAPKs, PI3K-Akt and NF-κB pathways and facilitates osteoclast differentiation on RANKL-primed pre-osteoclasts. KMUP-1 treatment significantly decreases the aforementioned signaling pathways enhanced by *Pg*LPS, which is consistent with our previous studies and may explain its protective function in the suppression of *Pg*LPS-mediated osteoclast formation.

Gum inflammation and pathogen accumulation are two primary risk factors associated with periodontal diseases. In this study, we used two established experimental periodontitis models in rats to evaluate KMUP-1’s protective effects on alveolar bone structure. *Pg*LPS injection directly induces periodontal inflammation, representing the acute phase of periodontitis progression. In the other model, ligature placement around the cervical region of molar teeth demonstrates a chronic and multi-factory periodontal diseases progression (de Molon et al., 2018). We successfully conducted these two animal models and showed significant periodontitis induction and substantial alveolar bone resorption as demonstrated by an increase of CEJ-ABC distance and trabecular separation as well as a decrease of BV/TV and trabecular thickness either by *Pg*LPS or ligature placement (Figures 6, 7). The KMUP-1-treated group exhibited an anti-bone resorption effect possibly due to the inhibition of osteoclastogenesis and induction of osteoblastogenesis, as demonstrated in our previous study (Liou et al., 2015). Bone resorption is regulated by the process of osteoclastogenesis, which is accelerated by inflammatory stimulation. Thus, anti-inflammatory adjunct therapies can contribute to the attenuation of inflammatory bone loss (i.e., bone loss due to periodontitis). All these pathological phenotypes of periodontitis were reduced by KMUP-1 treatment, suggesting that KMUP-1 exhibits a broad spectrum of effects in modulating chronic and acute gum inflammation. Therefore, the reduction of osteoclast differentiation after treatment of KMUP-1 may explain the therapeutic effect of KMUP-1 on experimental periodontitis.

**FIGURE 8:**
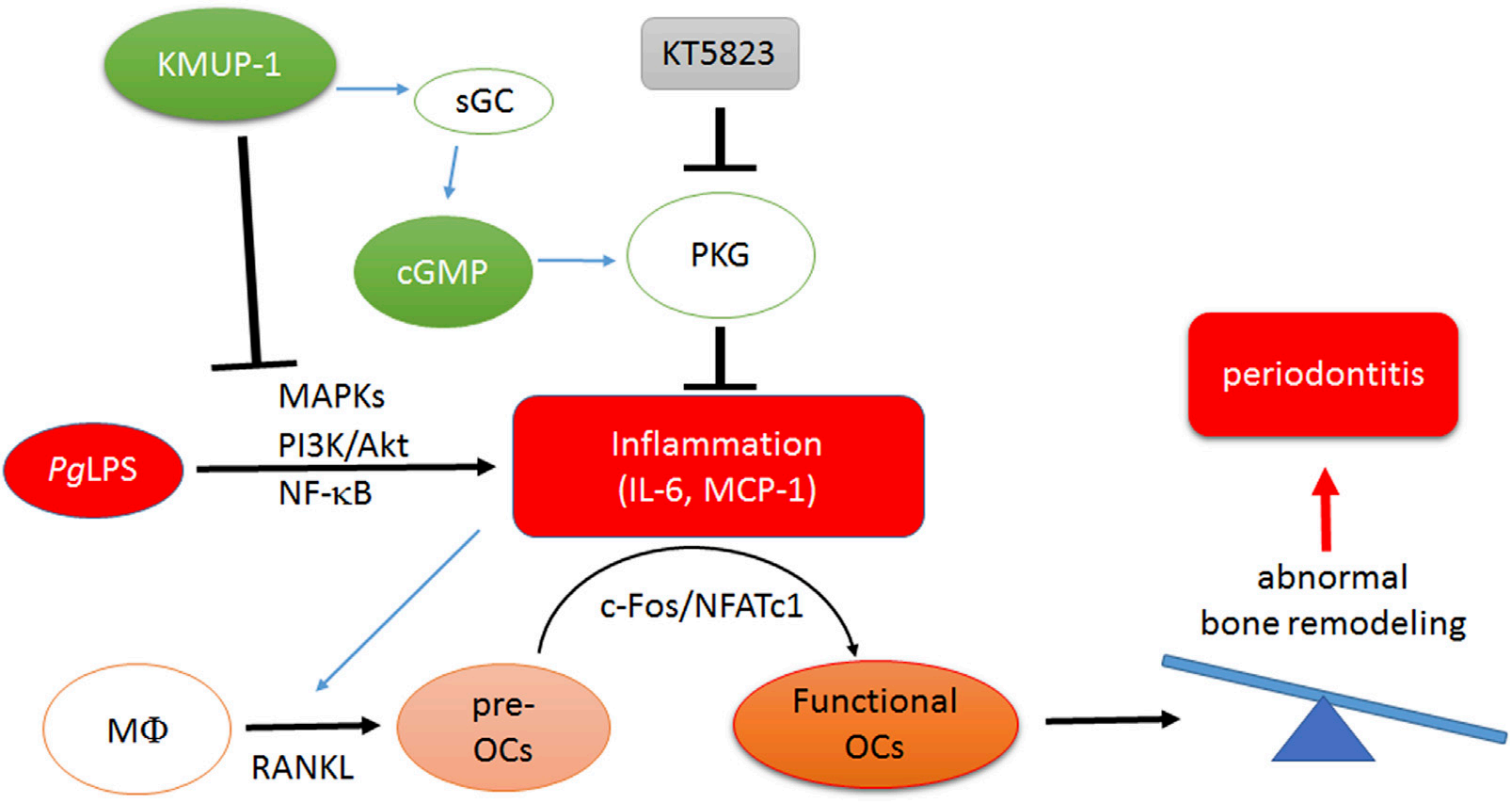
Schematic illustration of molecular mechanism underlying inhibition of *Pg*LPS-induced periodontitis by treatment of KMUP-1. *Pg*LPS triggers pro-inflammatory reactions and thus promotes maturation of osteoclasts. Amplification of osteoclastogenesis leads to imbalance of bone remodeling which aggravates periodontitis progression. KMUP-1, through elevation of the cGMP-PKG pathway and reduction of pro-inflammatory signaling pathways, suppresses *Pg*LPS-induced osteoclast differentiation thereby diminishing functional osteoclast maturation.

Taken together, we demonstrated that KMUP-1 attenuates pro-inflammatory osteoclast phenotypes and osteoclastogenesis elicited by *Pg*LPS in RANKL-primed RAW264.7 cells through a cGMP/PKG-dependent pathway. Moreover, reduction of pro-inflammatory signaling pathways and cytokine expression and downregulation of crucial regulators of osteoclasts, c-Fos and NFATc1, by KMUP-1 may explain its protective functions on *Pg*LPS-induced osteoclast maturation and alveolar bone loss in rat models (Figure 8).

## Conclusion

In summary, this is the first report demonstrating that KMUP-1 protects against *Pg*LPS- and ligature-induced periodontitis, at least partly, through its anti-inflammatory actions. Our results provide substantial evidence for conducting potential trials with KMUP-1 in companion animals (i.e., dog or cat) and humans for oral care as an adjunctive therapy since it did not exhibit any foreseeable adverse effect. Therefore, we conclude that KMUP-1 could be a potential therapeutics for inflammatory gum diseases.

## Data Availability

The raw data supporting the conclusions of this article will be made available by the authors, without undue reservation.
